# Supplementary material for: Influenza Infection in Ferrets with SARS-CoV-2 Infection History
Source: Microbiol Spectr. 2022 Oct 27;10(6):e01386-22. doi: 10.1128/spectrum.01386-22 (PMC9784775; doi:10.1128/spectrum.01386-22)
Supplement: Supplemental file 1 — Table S1. Download spectrum.01386-22-s0001.pdf, PDF file, 0.1 MB [file spectrum.01386-22-s0001.pdf]

Supplementary material

Table S1. Baseline serology of the ferrets included in this study.

Group	CHV	FCOV	CCV	Aleutian	Reference
**SARS-CoV-2**	150	150	450	<100	≤100
	100	150	800	<100	≤100
	100	200	>800	<100	≤100
	150	250	>800	<100	≤100
	100	200	>800	<100	≤100
	100	≤100	225	<100	≤100
**H1N1**	<100	<100	225	<100	≤100
	300	150	500	100	≤100
	300	150	300	200	≤100
	150	100	300	≤100	≤100
**SARS-CoV-2/H1N1**	100	≤100	100	100	≤100
	100	150	325	<100	≤100
	150	150	325	<100	≤100
	150	200	175	<100	≤100
	250	400	500	200	≤100
	100	<100	200	<100	≤100
	150	<100	750	100	≤100
	≤100	100	150	<100	≤100
	100	200	150	<100	≤100
	150	100	350	100	≤100
	150	<100	650	<100	≤100

**Mock-infected**	100	<100	100	<100	≤100
	150	100	150	<100	≤100
	200	150	650	100	≤100

Note: Data is expressed in optical density values obtained by functional ELISA assay from ferret sera for ferret coronavirus (FCOV), enteric coronavirus (CCV), Aleutian disease and canine herpes virus (CHV).
